# Development of Cellulosic Paper-Based Test Strips for Mercury(II) Determination in Aqueous Solution

**DOI:** 10.1155/2018/3594020

**Published:** 2018-11-01

**Authors:** Shoujuan Wang, Zhen Xu, Yongyi Fang, Zhongming Liu, Xin Zhao, Guihua Yang, Fangong Kong

**Affiliations:** ^1^State Key Lab of Biobased Material and Green Papermaking, Key Laboratory of Pulp and Paper Science and Technology Ministry of Education, Qilu University of Technology (Shandong Academy of Sciences), Jinan, Shandong 250353, China; ^2^Xuancheng Product Quality Supervision and Inspection Institute, Xuan Cheng, Anhui 242000, China

## Abstract

Titration method (dropping-on method) was introduced as an efficient approach for determining the mercury ion (Hg^2+^) concentration in aqueous solution by using fabricated cellulosic paper-based test strips. In this study, dithizone used as a recognition reagent was physically loaded on cellulosic paper-based test strips for Hg^2+^ selective recognition. The sensing mechanism was established on the spectral absorption rate of the coordination compound that was formed by dithizone and Hg^2+^ under strong acidic conditions. The calibration curve was obtained by the absorbency of Hg^2+^-dithizone complexes from different Hg^2+^ concentration solutions, and the correlation coefficient (*R*^2^) reached 0.9971. The detection range of the test trip for Hg^2+^ was obtained at 0.1 *μ*g/mL to 30 *μ*g/mL. Moreover, these superior cellulosic paper-based test strips have a rapid color-forming time (1.5 min) and low volume demand (3.7 *μ*L samples at 0.0127 g/L dithizone recognition concentration). This portable paper-based test strip can give potential applications for field screening or on-site semiquantitative analysis.

## 1. Introduction

Mercury is one of the most toxic elements to human health, causing several symptoms related to polyneuropathy mercurialis, neurasthenia, etc. [1]. Mercury ions can persistently exist in water environments and accumulate easily in organisms. Moreover, it has high toxicity, even in low concentrations [2]. Therefore, mercury pollution is an important issue that demands an ongoing development of analytical procedures to ensure its reliable determination [3, 4]. To fulfill the demands of the environmentally sustainable oriented goal for determining mercury in various matrices and at various levels of concentration, an essential tool is needed to assure accuracy and establish the traceability of the measured results. A lot of approaches have been used to develop optical or visual disposable sensors for mercury ion determination. The most common approaches are methods with spectrometric detectors [5–7]. Meanwhile, several compounds have been shown as selective for metal analysis and used as receptors for the design of sensing systems, ranging from alloys and amalgam [8] and synthetic metal ionophores [9, 10] to biological receptors [9, 11].

A fluorescence-based paper test strip is developed by using *N*-alkylaminopyrazole ligands for mercury detection in water, and a linear range from 10 *μ*g/mL to 100 *μ*g/mL mercury ion concentration ([Hg^2+^]) was achieved [2]. Meanwhile, pyrazole-derived ligands have been determined to be suitable for the coordination of metal ions [12–14]. A polyester sheet with a sensing area containing plasticized polyvinyl chloride (PVC) was made by incorporating tetraarylborate salt as a selective recognition reagent and a porphyrin proton-selective fluoroionophore as the optical transducer for Hg^2+^ [15]. Later, Yallouz et al. incorporated cuprous iodide (Cu_2_I_2_) carboxymethyl cellulose membranes on paper for Hg^2+^ in fish samples [16]. Shi and Jiang used a pH indicator color for Hg^2+^ in waters with a detection limit of 0.2 ng/mL under the inhibition of the different types of enzymes [17]. Capitán-Vallvey et al. developed an irreversible optical test strip for mercury determination based on an ion-exchange mechanism [18]. A circular sensing zone containing the essential reagents was used to produce a selective response for Hg^2+^ and consisted of plasticized PVC incorporating the cation-selective neutral ionophore. Chandio et al. also developed a PVC membrane ion selective ported to determine the Hg^2+^ in waste water [19]. Gur'eva et al. developed a test method for the mercury(I, II) determination by putting an intensely colored insoluble compound on a polycaproamide membrane surface in weak acid solutions depending on the reaction between mercury(I, II) and 5-chloro-2-hydroxy-3-[(tetrahydro-2,4-dithioxo-1,3-thiazin-5-yl) azo] benzenesulphonic acid [20]. However, most of these methods require expensive chemicals and instruments, and the treating procedure is complex and not easy to handle. In addition, most of these test strips are based on the plastic membrane technology. The used test strips are not reusable and biodegradable after disposal. Also, as the diffusion of Hg^2+^-containing solution in the plastic membrane is very slow even there are plasticizers in the membrane, the color-forming time usually needs a few minutes, even a few hours, which is not suitable for on-site screening analysis. Therefore, great emphasis still needs to be placed on the development of a low cost and convenient method for mercury determination.

Dithizone, as a kind of color-forming agent, has low cost and efficiency advantages, which can produce colored complexes between the color-forming agent in the paper strip and Hg^2+^ in the sample [21]. Based on the reaction between the dithizone and Hg^2+^ in the trichloromethane under strongly acidic conditions, the paper-based test strip for determining the concentration of Hg^2+^ in aqueous solutions could be developed. The test strip reacted rapidly with Hg^2+^ could lead to color formation of the test strip. Subsequently, the test color liquid diffused in indefinite directions due to liquid diffusivity. For the dipping-in method, the absorbency value continued to increase with the reaction time, and it was difficult to determine the result of color forming due to the reaction of the test strip and Hg^2+^ or liquid diffusivity.

The aim of this work is to introduce an efficient and simple approach to fabricate cellulosic paper-based test strips by physically impregnating paper strips in dithizone-containing solution to load dithizone into strips for determining the Hg^2+^ in aqueous solution. This preparation of the dithizone-loaded test strip is very simple and easily operated using biodegradable materials, paper strips, at low cost compared with other plastic-based test strips. In this study, dithizone is used as a recognition reagent to achieve selective recognition by Hg^2+^ with dithizone under strong acidic conditions. The calibration curve and pertinence are obtained by the absorbency from different concentrations of Hg^2+^. Furthermore, all the experiments are performed towards the detection linear range of the test strip system and superior test conditions of cellulose paper-based test strips.

## 2. Experimental

### 2.1. Materials

The paper sample used for the test strip was quantitative filter paper of medium porosity, which was obtained from Fisher Scientific (Jinan, China). Dithizone was obtained from Sigma-Aldrich (Jinan, China). Mercury solutions with various concentrations were freshly prepared by dissolving a certain amount of mercury nitrate in nitric acid solution with pH 2.0. Other solutions, including mercury ions (Hg^2+^), potassium ions (K^+^), calcium ions (Ca^2+^), sodium ions (Na^+^), manganese ions (Mn^2+^), magnesium ions (Mg^2+^), copper ions (Cu^2+^), barium ions (Ba^2+^), aluminum ions (Al^3+^), iron ions (Fe^3+^), zinc ions (Zn^2+^), phosphate ions (PO_4_^3-^), chloride ions (Cl^−^), and sulfate ions (SO_4_^2−^), were prepared by using analytical reagent-grade chemicals and nitric acid solution with pH 2.0. The pH of solutions was adjusted by adding either diluted sodium hydroxide (NaOH) or nitric acid (HNO_3_) solution. The spiked water sample of Hg(II) was prepared by adding Hg(II) into tap water with its pH adjusted to 2.0.

### 2.2. Apparatus

A UV-Vis spectrophotometer (UV-2550, Shimadzu, Nagoya, Japan) was used to determine the absorption of the colored complex solutions. A reflect spectrophotometer (i1Basic Pro2, X-Rite, Agilent, Foster City, California, USA) was used for the measurement of color intensity formed on the test strip.

### 2.3. Preparation of Cellulosic Paper-Based Test Strip

The dithizone was dissolved in trichloromethane (Fisher Scientific, Jinan, China) with a concentration of 0.0127 g/L. The filter paper was then immersed into this solution for 2 min at room temperature. The filter paper with dithizone was dried at 50°C in a nitrogen protected environment. The dried filter paper was then cut into 5 cm × 1 cm strips, sealed in a plastic bag, and kept until further use. The paper strip immersed in 0.0127 g/L dithizone solution had a 0.33 mg/cm^2^ dithizone loading amount.

## 3. Methods

### 3.1. Spectral Reflectivity Measurement

The Hg^2+^ solution was dropped on the paper-based test strip. After the color-forming reaction finished, the color intensity developed on the test strip was measured using the reflect spectrophotometer at a fixed wavelength of 490 nm.

The color forming depended on the dithizone reaction with mercury(II) and the coordination compound (dithizone-mercury complex) was formed, and the linear relationship between the absorbency value and different Hg^2+^ concentrations was tested by spectrophotometer at a fixed wavelength of 490 nm [22].

### 3.2. Hg(II) Determination Using UV-Vis Spectroscopy Method

A 250 mL mercury solution with 10 *μ*g/mL concentration was put into a separatory funnel (500 mL) by adding 1 mL sodium sulfite (20%). After mixing, 10 mL dithizone-trichloromethane solution was added, and the solution layered after 1 min. The mercury solution after contacting with the dithizone was dissolved in the trichloromethane and was transferred to a cuvette of the spectrophotometric measurements. A UV-Vis spectrophotometer (UV-2550, Shimadzu, Nagoya, Japan) was used to measure the absorbance value of the dithizone-mercury complex in the solution [22]. Then, the concentration of Hg(II) was calculated based on the calibration curve obtained in this study:

(1) $$\begin{array}{c} Y=0.0558X+0.0391,\\ R^{2}=0.9932, \end{array}$$

where *Y* is the absorbance value and *X* is the mercury ion concentration (*μ*g/mL).

## 4. Results and Discussion

### 4.1. Development of Paper-Based Test Strip

Due to the high specific surface area and plenty of capillary pores, paper sheets, such as filter paper, have high absorbability of inorganic or organic liquids [23]. By loading the effective chemicals onto a paper sheet, a paper-based test strip specific for the determination of Hg(II) concentration is expected to be portable, user-friendly, and handy. From this viewpoint, the selection of a suitable reagent to extract the Hg(II) content puts emphasis on the following considerations. This paper-based test strip would have specific features of (i) giving a color response so that the test strip, to be used with a color chart or miniature device, can quantitatively determine the Hg(II) concentration in the field screening or on-site semiquantitative analysis without excessive laboratory instruments and (ii) having a selectivity for Hg(II) so that the sample pretreatment steps would be minimized. Because of the high stability constant of the Hg(II)-dithizone complex, the dithizone formed a much more stable complex with Hg(II). The extraction process of Hg(II) was performed using dithizone with a high selectivity to avoid any interfering elements in the extraction process.

**Figure 1:**
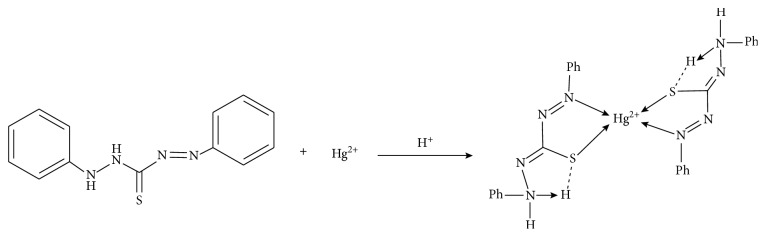
The detection principle for the mercury assay.

The detection principle for the mercury assay of this test strip method is shown in Figure 1 [24]. Dithizone is one of the foremost extractants that is recognized as a sensitive reagent for the determination of Hg(II) in acidic media. It is capable of forming primary and secondary dithizonates with Hg(II) [25, 26]. Meanwhile, due to a higher absorptivity coefficient and solubility in the organic phase, the primary mercury-dithizonate chelate is preferred in the spectrophotometric determination as well.

The dissolved diatomic mercury ions, Hg(II), are the common form in aqueous solution. The Hg(II) could react with dithizone under acidic condition, and the complex is colored. In addition, the colored complex and the concentration of the Hg(II) ion are in accordance with the Lambert–Beer law:

(2) $$\begin{array}{c} A=\mathrm{lg}\left(\frac{1}{T}\right)=kbc\text{,} \end{array}$$

where *A* is the absorbency (L/(mol∗cm)), *T* is the transmissivity (°C), and *b* and *c* are the concentration of the light-absorbing material (mol/L) and the thickness of the absorbed layer (cm), respectively.

**Figure 2:**
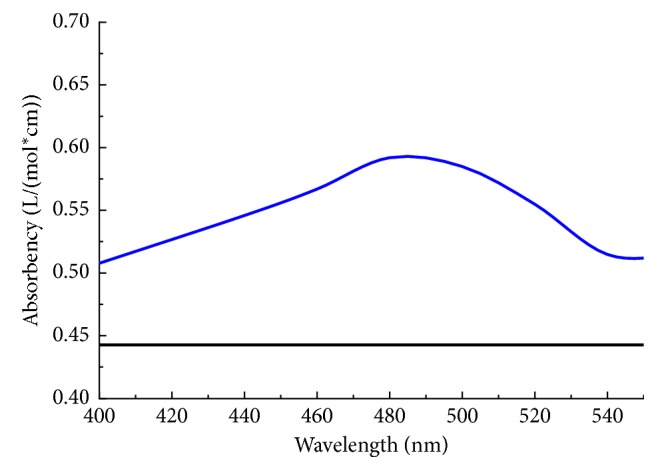
Characteristic absorption of the mercury-dithizone complexes in aqueous solution.

Figure 2 shows the characteristic absorption of the mercury-dithizone complexes in aqueous solution. The absorbency gradually increased as the wavelength increased to 490 nm. Afterwards, the absorbency gradually decreased as the wavelength continued to increase. The results showed the maximum absorption peak of this Hg(II)-dithizone complex was at 490 nm. Subsequently, the measurement of spectra reflectivity was measured at 490 nm. The apparent molar absorptivity at 490 nm was 0.6 L·mol^−1^·cm^−1^.

**Figure 3:**
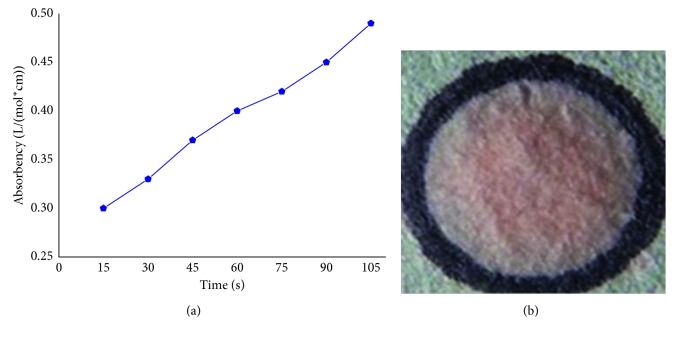
Different images of dipping-in method and dropping-on method: (a) the linear relationship between absorbency value and different dipping time of test strip and Hg^2+^ by dipping-in method and (b) test strip with marker circle and mercury-dithizone colored complexes forming by dropping-on method.

Figure 3(a) shows the linear relationship between the absorbency value and different reaction times of the test strip and Hg^2+^ via the dipping-in method, which is that the paper-based test strip is dipped directly into the Hg^2+^-containing solution firstly and then pulled it out from the solution to measure the absorbency. In this process, the test strip reacted rapidly with Hg^2+^ in solution, which led to color formation of the test strip. However, in this process, the Hg^2+^ contained in solution can continuously react with dithizone which loaded in the paper-based test strip, which caused the color absorbency of mercury-dithizone complexes formed on paper-based test strip increase with the prolongation of dipping time, as shown in Figure 3(a). The dipping-in time of the test strip needs to be strictly and accurately controlled if this dipping-in method was used in determining Hg^2+^ concentration, which is very difficult to operate by hand especially in on-site screening analysis. To solve this issue, the dropping-on method was chosen to use. In this method, certain amount of Hg^2+^-containing solution was dropped on the dithizone-loaded paper-based test strip. Subsequently, the absorbency of the test strip colored by mercury-dithizone complexes was measured using spectrophotometer. During the measurement of using this dropping-on method, it was found that the colored liquid (mercury-dithizone complexes) diffused in indefinite directions due to liquid diffusivity. For the dropping-on method, although the absorbency value does not continued to increase with the reaction time, it was still difficult to accurately determine the result of color forming due to nonuniformity of colored complexes on the paper strip due to the Hg^2+^ or liquid diffusivity.

To overcome this diffusivity phenomenon, a horizontal circle 9 mm in diameter was made on the paper-based test strip [27]. This marker circle served as a hydrophobic barrier to prevent the dropped mercury solution from dispersing, and the uniform color is displayed in Figure 3(b). The test strip shows a light green color at the absence of mercury solution. When a drop of test solution was placed on the test strip, as shown in Figure 3(b), the test strip turned into pink at the presence of mercury solution. According to a series of trials, 3.7 *μ*L was a suitable volume to be placed in the circle for preventing the mercury solution drop from dispersing and forming a uniform color. In addition, it should be noted that the dithizone loaded in this 90 mm circle test strip, 11.5 mmol, is much higher, 10 times higher, than that required for reacting with Hg^2+^, 1.5–3.5 mmol, in 3.7 *μ*L solution.

**Figure 4:**
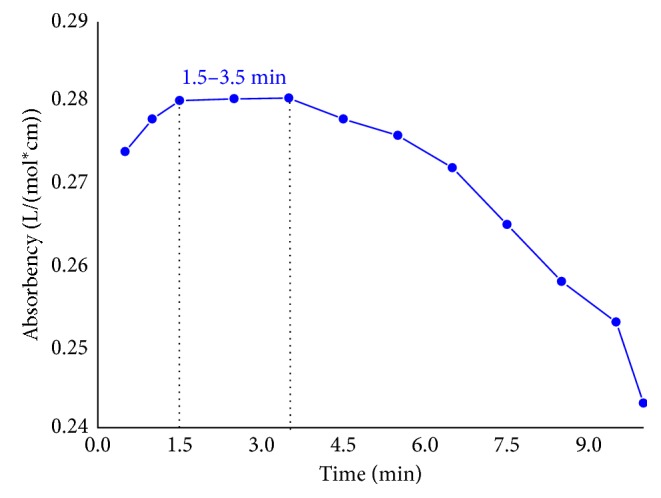
Absorbency of colored complexes formed using 3.7 *μ*L 30 *μ*g/mL Hg^2+^ solution and dithizone-loaded test strip at different color-forming time.

After solving this diffusivity phenomenon of mercury solution on the paper-based test strip, this dropping-on method could be used to determine the Hg^2+^ concentration in solution. In this method, the Hg^2+^-containing solution was quantificationally dropped on the surface of circled paper-based test strip, and then the Hg^2+^ in the dropped solution reacts with dithizone loaded in the test strip to form mercury-dithizone complexes which is colored in pink. The color intensity formed on the test strip is mainly affected by the reaction time between Hg^2+^ and dithizone, here, namely, color-forming time. The test strip absorbency (color intensity) served as a standard function of color-forming time. The absorbency, at 490 nm, of colored complexes formed using 30 *μ*g/mL mercury solution to react with dithizone-loaded test strip at different color-forming time (0.5 to 10 min) is presented in Figure 4. As shown in Figure 4, the absorbency of the test strip after dropping of Hg^2+^ solution gradually increased with color-forming time ranging from 0.5 min to 1.5 min, and then, the absorbency value tended to stabilize between 1.5 min and 3.5 min. The absorbency further reduced as the color-forming time prolonged beyond 3.5 min. The absorbency increase at color-forming time from 0.5 min to 1.5 min is due to formation of more mercury-dithizone complexes through the reaction of Hg^2+^ with dithizone. When the color-forming time reached 1.5 min, the absorbency stabilized, illustrating all the Hg^2+^ in the dropped solution reacted completely with dithizone loaded in test strip and formed the colored complexes, and this color intensity kept at a constant value for a while, up to 3.5 min. After 3.5 min, the decrease of absorbency is attributed to the water evaporation of the test strip. The maximum and most stable absorbency value (0.282) appeared at a 2.5 min color-forming time. The results showed that the reliable absorbency measurement of this test strip should be carried out in 1.5–3.5 minutes after dropping of Hg^2+^-containing solution on the test strip.

### 4.2. Calibration Curve

**Figure 5:**
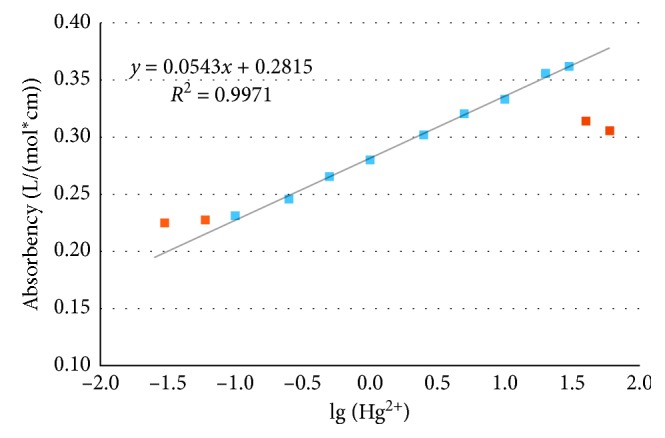
Working range of paper-based test strip (pH 2.0, color-forming time: 2.5 min).

To establish the calibration curve, mercury solutions with various concentrations were prepared and used (Figure 5). Under the optimum conditions, pH 2.0 and reaction time 2.5 min, a linear calibration curve was constructed for Hg(II) determination over the range of 0.1 *μ*g/mL to 30 *μ*g/mL. The correlation coefficient (R2) was 0.9971, which showed an acceptable linearity of the calibration curve. Through the data analysis presented in Figure 5, it is found that the upper and lower detection limits of this paper-based test strip method are 30 *μ*g/mL and 0.1 *μ*g/mL, respectively. Although this lower detection limit was not enough for detection of Hg^2+^ ions in drinking waters or lake waters, it could be used for determination of waste waters from industry. The paper-based test strip with much lower detection limit, suitable for detecting lake waters or drinking waters, is our another research work which is going and will be reported in detail in our another paper.

### 4.3. Selectivity of Paper-Based Test Strip

**Figure 6:**
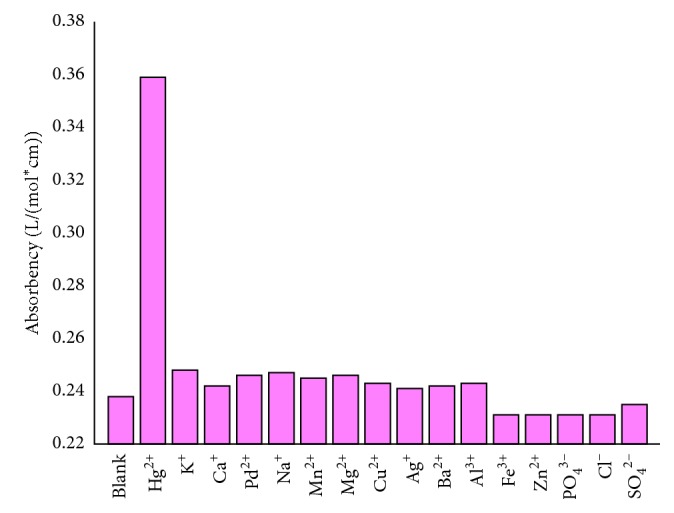
Absorbency of various metals in contact with the dithizone-loaded test strip; shown are the responses from blank (MilliQ water), 20 mg/L of Hg^2+^, K^+^, Ca^2+^, Na^+^, Mn^2+^, Mg^2+^, Cu^2+^, Ag^+^, Ba^2+^, Al^3+^, Fe^3+^, Zn^2+^, PO_4_^3−^, Cl^−^, and SO_4_^2−^, and the concentration of other ions were one thousand times of Hg^2+^ (color-developing time: 2.5 min).

In practice, Hg^2+^ cannot exist alone because large numbers of anions and cations also exist. However, the dithizone is effective on various ions. As shown in Figure 6, the test strips have absorbency for the different ions (Hg^2+^, K^+^, Ca^2+^, Na^+^, Mn^2+^, Mg^2+^, Cu^2+^, Ag^+^, Ba^2+^, Al^3+^, Fe^3+^, Pb^2+^, Zn^2+^, PO_4_^3−^, Cl^−^, and SO_4_^2−^). Furthermore, Hg^2+^ displayed excellent absorbability due to the test strips having better sensitivity and selectivity with the Hg^2+^ in the solution. Occasionally, Mn^2+^, Mg^2+^, Pb^2+^, Cu^2+^, Ag^+^, Cl^−^, and SO_4_^2−^ interfered a little with mercury [2], and according to the calibration curve, the deviations resulted from the interference of these ions are all less than 5%, which is acceptable usually for analysis [1, 9].

### 4.4. Verification of the Method with Spiked Water Samples

In this study, a high-efficiency test strip was developed in the circle with 3.7 *μ*L of the mercury solution by pipette, the color-forming time was fixed at 2.5 min, and compared with the reference method, UV-Vis spectroscopy method, by determining same mercury concentrations [18]. The mean values from three replicate samples and the standard deviations of these measurements are shown in Table 1. The measured concentrations of Hg(II) using the test strip were similar as these from the reference method (2.9 and 3.1; 6.2 and 6.0; 9.1 and 8.8; and 13.5 and 13.7). The results exhibited a good sensitivity and selectivity, and also the deviation of this test-strip method was similar as that of reference method, even better than the reference method at high Hg(II) concentration (0.05 and 0.05; 0.21 and 0.18; 0.25 and 0.30; and 0.19 and 0.32). The measured data denoted that the proposed method can be satisfactorily applied to the determination of trace Hg(II) in real samples.

## 5. Conclusions

A cellulosic paper-based test strip for specially determining Hg^2+^ was developed. The color-forming reagent dithizone was physically loaded into the quantitative filter paper by impregnation process, and the mercury-dithizone complexes have a characteristic magenta color at acidic condition. The high-efficiency test strip method was developed by dropping 3.7 *μ*L of the mercury solution via pipette on a circle test strip, and the color-forming time was fixed at 2.5 min. Furthermore, the paper-based test strips have a high selectivity to Hg^2+^ ion.

The developed method offered a good sensitivity and selectivity for the determination of Hg(II) in the concentration range of 0.1 *μ*g/mL to 30 *μ*g/mL. Although this lower detection limit was not enough for detection of Hg^2+^ ions in drinking waters or lake waters, it is enough to be used for detection of Hg^2+^ ion in waste waters from industry.

This paper-based test strip method, when applied to samples spiked with Hg^2+^ ions, gave accurate results in comparison with the conventional method for the determination of Hg^2+^. Therefore, it can be concluded that the paper-based test strip method developed in this study is a simple, effective, and reliable way of determining the Hg^2+^ ion concentration in aqueous sample.

## Figures and Tables

**Table 1:** Determination of Hg(II) concentration in a spiked tap water sample based on the test strip method.

Sample ID	Test strip method		Reference method	
	[Hg^2+^] (*μ*g/mL)^*∗*^	Standard deviation (*μ*g/mL)	[Hg^2+^] (*μ*g/mL)^*∗*^	Standard deviation (*μ*g/mL)
1	2.9	0.05	3.1	0.05
2	6.2	0.21	6.0	0.18
3	9.1	0.25	8.8	0.30
4	13.5	0.19	13.7	0.32

^*∗*^Average value (*n*=3).

## Data Availability

The data used to support the findings of this study are available from the corresponding author upon request.

## References

[1] Chen J., Li Y., Zhong W., Wang H., Zhang P., Jiang J. (2016). A highly selective fluorescent and colorimetric chemosensor for Hg^2+^ based on a new rhodamine derivative. *Analytical Methods*.

[2] Kolb M., Bahadir M., Teichgräber B. (2017). Determination of chemical oxygen demand (COD) using an alternative wet chemical method free of mercury and dichromate. *Water Research*.

[3] Aragay G., Montón H., Pons J., Fontbardía M., Merkoçi A. (2012). Rapid and highly sensitive detection of mercury ions using a fluorescence-based paper test strip with an N-alkylaminopyrazole ligand as a receptor. *Journal of Materials Chemistry*.

[4] Richard J. H., Biester H. (2016). Mercury removal from contaminated groundwater: performance and limitations of amalgamation through brass shavings. *Water Research*.

[5] Deeds D. A., Ghoshdastidar A., Raofie F., Guérette É. A., Tessier A., Ariya P. A. (2015). Development of a particle-trap preconcentration-soft ionization mass spectrometric technique for the quantification of mercury halides in air. *Analytical Chemistry*.

[6] Fashi A., Yaftian M. R., Zamani A. (2017). Electromembrane extraction-preconcentration followed by microvolume UV–Vis spectrophotometric determination of mercury in water and fish samples. *Food Chemistry*.

[7] Ma K., Li X., Xu B., Tian W. (2014). A sensitive and selective “turn-on” fluorescent probe for Hg^2+^ based on thymine–Hg^2+^–thymine complex with an aggregation-induced emission feature. *Analytical Methods*.

[8] Tao H., Lin Y., Yan J., Di J. (2014). A plasmonic mercury sensor based on silver–gold alloy nanoparticles electrodeposited on indium tin oxide glass. *Electrochemistry Communications*.

[9] Farzin L., Shamsipur M., Tabrizi M. A. (2015). Biomagnetic separation and pre-concentration of trace amounts of Hg^2+^ in biological samples based on T-rich oligonucleotide modified magnetic beads. *Analytical Methods*.

[10] Silwana B., Van D. H. C., Iwuoha E., Somerset V. (2014). Amperometric determination of cadmium, lead, and mercury metal ions using a novel polymer immobilised horseradish peroxidase biosensor system. *ournal of Environmental Science and Health, Part A*.

[11] Scheuhammer A., Braune B., Chan H. M. (2015). Recent progress on our understanding of the biological effects of mercury in fish and wildlife in the Canadian Arctic. *Science of The Total Environment*.

[12] Cegłowski M., Schroeder G. (2015). Removal of heavy metal ions with the use of chelating polymers obtained by grafting pyridine–pyrazole ligands onto polymethylhydrosiloxane. *Chemical Engineering Journal*.

[13] Tomás-Gamasa M., Serdjukow M. S. S., Su M. S. M., Müller M., Carell P. T. (2015). Removal of heavy metal ions with the use of chelating polymers obtained by grafting pyridine–pyrazole ligands onto polymethylhydrosiloxane. *Angewandte Chemie International Edition*.

[14] Pettinari C., Tăbăcaru A., Galli S. (2016). Coordination polymers and metal–organic frameworks based on poly(pyrazole)-containing ligands. *Coordination Chemistry Reviews*.

[15] Cano-Raya C., Fernández-Ramos M. D., Gómez-Sánchez J., Capitán-Vallvey L. F. (2006). Irreversible optical sensor for mercury determination based on tetraarylborate decomposition. *Sensors and Actuator B: Chemical*.

[16] Yallouz A. V., Calixto D. C. R., Paciornik S. (2000). A low-cost non instrumental method for semiquantitative determination of mercury in fish. *Fresenius’ Journal of Analytical Chemistry*.

[17] Shi G. Q., Jiang G. (2002). A dip-and-read test strip for the determination of mercury(II) ion in aqueous samples based on urease activity inhibition. *Analytical Sciences*.

[18] Capitán-Vallvey L. F., Raya C. C., López E. L., Ramos F. (2004). Irreversible optical test strip for mercury determination based on neutral ionophore. *Analytica Chimica Acta*.

[19] Chandio Z., Talpur F., Khan H., Afridi H., Khaskheli G., Mughal M. (2014). On-line preconcentration and determination of ultra trace amounts of mercury using surfactant coated alumina modified by dithizone with cold vapor atomic absorption spectrometry. *RSC Advances*.

[20] Gur’eva R. F., Savvin S. B., Mikhailova A. V. (2003). Sorption and determination of vanadium(IV, V) and Mercury(I, II) as their colored complexes of organic reagents. *Journal of Analytical Chemistry*.

[21] Sedghi R., Kazemi S., Heidari B. (2017). Novel selective and sensitive dual colorimetric sensor for mercury and lead ions derived from dithizone-polymeric nanocomposite hybrid. *Sensors and Actuators B: Chemical*.

[22] Yamamura S. S., Sikes J. H. (1966). Use of Citrate-EDTA masking for selective determination of iron with 1, 10-phenanthroline. *Analytical Chemistry*.

[23] Carrera S., Santiago G., Vega M. (2016). Spectrophotometric determination of dithizone–mercury complex by solid phase microextraction in micropipette tip syringe packed with activated carbon xerogel. *Microchemical Journal*.

[24] Zhang Z., Li J., Song X., Ma J., Chen L. (2014). Hg^2+^ ion-imprinted polymers sorbents based on dithizone–Hg^2+^ chelation for mercury speciation analysis in environmental and biological samples. *RSC Advances*.

[25] Mudasir M., Karelius K., Aprilita N. H., Wahyuni E. T. (2016). Adsorption of mercury(II) on dithizone-immobilized natural zeolite. *Journal of Environmental Chemical Engineering*.

[26] Zhang D., Sun M., Zou L. (2016). *A Review on Spectrometer of Pb(II) in Water*.

[27] Satoh A. Y., Trosko J. E., Masten S. J. (2007). Methylene blue dye test for rapid qualitative detection of hydroxyl radicals formed in a fenton’s reaction aqueous solution. *Environmental Science and Technology*.

